# Exploration of experiences in therapeutic groups for patients with severe mental illness: development of the Ferrara group experiences scale (FE- GES)

**DOI:** 10.1186/1471-244X-13-242

**Published:** 2013-10-01

**Authors:** Rosangela Caruso, Luigi Grassi, Bruno Biancosino, Luciana Marmai, Luciano Bonatti, Maria Moscara, Marco Rigatelli, Catherine Carr, Stefan Priebe

**Affiliations:** 1 Department of Behavior and Communication, Section of Psychiatry, University of Ferrara, Ferrara, Italy; 2 Department of Mental Health and Drug Abuse, Ferrara Health Agency, Ferrara, Italy; 3 Department of Mental Health and Drug Abuse, Bologna Health Agency, Bologna, Italy; 4 Department of Neuroscience, Section of Psychiatry, University of Modena and Reggio Emilia, Modena, Italy; 5 Unit for Social and Community Psychiatry, Queen Mary University of London, London, UK

**Keywords:** Group therapy, Patient experiences, Severe mental illness, Community mental health care

## Abstract

**Background:**

Group therapies are routinely provided for patients with severe mental illness. The factors important to the group experience of patients are still poorly understood and are rarely measured. To support further research and practice, we aimed to develop a questionnaire that captures how patients experience groups within a community mental health context.

**Methods:**

An initial pool of 39 items was conceptually generated to assess different aspects of group experiences. Items were completed by 166 patients with severe mental illness attending group therapies in community mental health services in Italy. Patients with different psychiatric diagnoses who attended at least 5 group sessions were included. An exploratory factor analysis was used to identify different dimensions of group experiences and to reduce the number of items for each dimension.

**Results:**

The resulting questionnaire has five subscales: 1) sharing of emotions and experiences, 2) cognitive improvement, 3) group learning, 4) difficulties in open expression and 5) relationships. Each subscale has 4 items. The scale and sub-scales have good internal consistency.

**Conclusions:**

The Ferrara Group Experiences Scale is conceptually derived and assesses dimensions of group experience that are theoretically and practically relevant. It is brief, easy to use and has good psychometric properties. After further validation, the scale may be used for research into patient experiences across different group therapy modalities and for evaluation in routine care.

## Background

Since the introduction of therapeutic communities in the 1940s, group treatments have become a common part of care for patients with severe mental illness [1-7]. Group treatments are widely provided in psychiatric settings and generally considered as a routine therapeutic intervention [8-11]. It has been suggested that group experiences can be a powerful agent of change and efficacy has been demonstrated across a range of approaches including cognitive-behavioural therapy [8], integrated psychological therapy [9], social skills training [10], cognitive remediation [11], and others [12-17].

Several processes have been proposed to explain the mechanisms of group therapy [1,4,5,18,19]. Yalom’s studies of the essential mechanisms for change common to group treatments [20,21] identified eleven factors consisting of: 1) instillation of hope, 2) universality, 3) imparting of information, 4) altruism, 5) the corrective recapitulation of the primary family group, 6) development of socializing techniques, 7) imitative behaviour, 8) interpersonal learning, 9) group cohesiveness, 10) catharsis, and 11) existential factors. Bloch and Crouch [22] later formulated a model that proposed *self-disclosure* and *self-understanding* as predictors of change. A recent study suggests that Yalom’s factors do not have equal importance to patients and therapists. Cohesiveness, universality and self-understanding were indicated as most important in the group experience [23].

A number of scales have been developed to explore different aspects of group treatment. Many have been designed to specifically evaluate the presence of Yalom’s 11 factors [6,7,23-27]. More recently, Dierick and Lietaer [28], developed a questionnaire to investigate therapeutic factors across an extensive range of group therapies from the client’s perspective. Participants were diverse and represented inpatients and outpatients, as well as students and trainee psychotherapists. Therapeutic approaches included client centred/experiential, Gestalt, psychoanalytic, behavioural, drama and body therapies. The questionnaire comprised of 133 items and 28 basic scales. Questions were written from the perspective of the patient’s experience, and focused primarily on helpful or instructive experiences. The method used a series of multivariate analyses to define the links between therapeutic factors and their degree of interconnectedness. Two different dimensions were proposed to account for the therapeutic factors intervening in groups. These two dimensions were interpreted as “Relational Climate” and “Psychological Work”.

Despite the comprehensiveness of this questionnaire, its use may be limited in a community mental health setting as the number of items makes the scale difficult to implement in routine clinical evaluation and items were derived from a mostly non-clinical sample. The questionnaire focuses only on items experienced as helpful or instructive and therefore does not explore experiences and feelings that were perceived as less therapeutic or detrimental.

Despite the body of research regarding therapeutic mechanisms in group treatment, a valid, brief and robust scale that explores the core experiences of patients (therapeutic or not) during a single session or course of group therapy does not currently exist in community psychiatry. The purpose of this study was to develop and perform a primary validation of a questionnaire to assess the main subjective experiences of patients with severe mental illness attending group therapy. The questionnaire was designed to be specific to community mental health services, short and easy to administer and to capture different but theoretically meaningful dimensions of experiences across a range of group interventions and community settings.

## Methods

### Setting

The study took place in community mental health services of three provinces in North-east Italy. Settings included a residential unit in Ferrara, providing short-to medium-term care for patients with acute and subacute psychiatric conditions, and two psychiatric day-hospitals in Bologna and Modena. A range of group activities are regularly offered within these settings and patients are invited and encouraged to participate in all groups.

### Group intervention

Group interventions were scheduled on weekly basis. All groups lasted for 90 minutes, except the psychodynamic group (60 minutes) and had the following characteristics:

• Group psychodynamic psychotherapy provided opportunities for each member to learn about themselves and their interpersonal and intra-psychic functioning, by creating a safe atmosphere that encouraged interaction and invited reflection.

• Psychosocial rehabilitation groups (e.g. newspaper reading groups) had a more practical focus, whilst pursuing the improvement of patients’ cognitive and social skills.

• Psycho-educational groups (such as a “wellness group”) provided patients with information about their psychiatric disorders, pharmacological treatments and healthy living.

• Expressive groups (such as arts and music therapy groups) gave patients the opportunity to be creative and encouraged self-expression.

• Body oriented groups included a relaxation group and psycho-motor group.

• “Other” groups represented groups where patients could discuss issues and raise concerns.

### Generation of the item pool

The questionnaire was developed in several steps. At each stage, decisions were discussed and made by the project team in Italy and then further checked with an expert in scale development (SP) to reduce bias.

An initial item pool was generated by focus groups held in three community mental health services between September-October 2009 (Ferrara, coordinating centre; Modena and Bologna). Each group comprised of clinicians from different professional backgrounds that commonly participate in the management of groups (Ferrara: 2 psychiatrists, 5 psychiatric nurses, 2 social workers, 1 psychologist; Modena: 2 psychiatrists, 2 psychiatric nurses, 2 rehabilitation therapists, 1 psychologist; Bologna: 1 psychiatrist, 5 psychiatric nurses, 2 rehabilitation therapists and 1 psychologist). Each focus group met four times. The task of each meeting was to generate a complete list of common therapeutic group experiences which resulted in a list of 25 group experiences from the Ferrara group, 21 from Modena and 26 from Bologna.

The items were then examined by the coordinating centre in Ferrara and grouped into common dimensions. Four conceptual dimensions resulted: relational aspects, expression of emotions and mentalization, group learning, and cognitive experiences. Four statements that most clearly represented each dimension were selected from the lists, to form a total of 16 experiences.

These were then transformed into specific questions with multiple questions for each statement to ensure comprehensive coverage (7 questions for relational aspects, 6 for expressions of emotions and mentalization, 6 for group learning, and 6 for cognitive improvement). This resulted in a pool of 25 questions which was circulated and approved by all the participating centres. Inverse items were then included (3 for relational aspects, 3 for expression of emotions, 4 for group learning and 3 for cognitive improvement) expanding the pool to 38 questions.

The questionnaire was read in a meeting with 12 psychiatric patients (5 with a diagnosis of schizophrenia, 3 with bipolar disorder, 3 with unipolar depression and 1 with borderline personality disorder) to verify that all items were clearly understandable and made sense. Any words that were unclear were exchanged with words suggested by the group. On the final reading, all questions were deemed understandable by the patients.

The resulting self-report instrument (Ferrara Group experiences Scale – FE-GES) assessed the main subjective experiences of participants in group therapy and the intensity this was felt. It consisted of 38 items evaluating the four hypothesised areas of *relationships* (e.g. “I was helped by others”, “I socialised with others”), *emotional expression and mentalization* (e.g. “I shared my personal experiences and my life problems”, “I met people who were experiencing the same problems as me”), *group learning* (e.g. “I understood the reasons for my behaviour”, “I understood better how I usually deal with my problems”) and *cognitive improvement* (e.g. “I paid attention to what others said”, “I could remember what was said”).

Items were rated on a 5-point Likert scale, from not at all (0), a little (1), enough (2), very (3) to a great deal (4). Items 3, 7, 11, 13, 16, 18, 22, 24, 26, 28, 32, 34 and 38 were inversely scored.

A further item was included, which asked the participant to rate the overall usefulness of the group. The questionnaire was written in Italian.

### Data collection

Psychiatric patients admitted to the participating services between January 1, 2010 and April 30, 2011 were screened for inclusion. Eligible patients were those with any psychiatric diagnosis according to ICD-10 criteria except for mental retardation (ICD-10 codes F70 to F79), who chose to take part in at least 5 group activity sessions of any modality and consented to participate in the study. Patients completed a psychiatric diagnostic interview according to ICD-10 criteria on admission. All patients were informed of the aim of the study and gave written informed consent. The study was conducted in line with the Code of Ethics of the World Medical Association (Declaration of Helsinki, 1964) and approved by the institutional review board of the University of Ferrara. Socio-demographic and clinical data (e.g. length of illness and number of hospitalizations) were collected. Prior to discharge from services, patients were asked to complete the questionnaire to rate their overall group experiences during the period of admission.

### Statistical analysis

Principle Components Analysis (PCA) of the 39 items was used to identify any underlying dimensions of this questionnaire. Both orthogonal (varimax) and oblique (direct-oblimin) rotations were examined using the FACTOR program [29]. Descriptive statistics were used to check the distribution of the data and to identify items with high values of skewness or kurtosis. Polychoric correlations were employed to account for the Likert scale response [30]. Inter-correlations between items were examined for particularly high or low correlations and the Kaiser-Meyer-Olkin measure was used to verify sampling adequacy. The strength of relationships between variables was assessed using Bartlett’s test of sphericity. The number of components to retain was first estimated by parallel analysis [31]. Rotations were examined for a range of 2–6 specified components around this estimate, and a criterion level of 0.4 was set to determine the importance of factor loadings [32]. Reliability of the rotated factor scores [33,34], Bentler’s [35] simplicity and Lorenzo-Seva’s [36] loading simplicity indices were then examined. Pattern and structure matrices were inspected in oblique rotations for items loading highly onto more than one component and the correlation matrix analysed to determine whether there were relationships between components. Low correlating and highly skewed items were systematically removed and the analysis re-run. The final solution was selected based upon criteria of explanation of a good proportion of the variance, balance of loading onto components and conceptual similarity. Items with the highest loadings onto components were selected and analysed for internal consistency using Cronbach’s α in PASW Statistics (v.18). A criteria of α > .7 was set as a minimum [37].

## Results

### Sociodemographic and clinical characteristics of patients

During the study period, 191 patients were admitted across the 3 participating centres, of which 166 were eligible and consented to participate. Table 1 displays rates of participation in each group.

**Table 1:** Number of patients participating in groups by diagnosis

**Diagnosis (number of patients with that diagnosis and percentage in relation to the total)**		**Psychodynamic therapy**	**Psychosocial rehabilitation group**	**Psycho-educational group**	**Expressive group**	**Bodily mediated group**	**Others**
Schizophrenia	57 (34%)	44 (77%)	92 (56%)	16 (28%)	40 (70%)	29 (51%)	11 (19%)
Delusional Disorder	4 (2%)	3 (75%)	1 (25%)	1 (23%)	2 (50%)	1 (25%)	0 (0%)
Schizo-affective Disorder	10 (6%)	8 (80%)	7 (70%)	3 (30%)	7 (70%)	4 (40%)	4 (40%)
Bipolar Disorder	29 (18%)	26 (90%)	12 (41%)	8 (28%)	12 (41%)	12 (41%)	5 (17%)
Major Depression	27 (16%)	26 (96%)	13 (48%)	10 (37%)	13 (48%)	12 (44%)	5 (9%)
Anxiety Disorder	1 (1%)	1 (100%)	0 (0%)	0 (0%)	0 (0%)	0 (0%)	0 (0%)
Eating Disorder	12 (7%)	11 (92%)	1 (8%)	1 (8%)	0 (0%)	1 (8%)	2 (17%)
Personality Disorder	26 (16%)	25 (96%)	15 (58%)	8 (31%)	15 (58%)	11 (42%)	3 (12%)
Total	166 (100%)	144 (87%)	81 (49%)	47 (28%)	89 (54%)	70 (42%)	30 (18%)

The sample consisted of 58 men (35%) and 108 women (65%), with a mean age of 46.4 (± 12.2) years. Half of the participants had never married (83; 50%); 44 were married (27%), 31 divorced (19%) and 8 widowed (5%). Most were unemployed (120; 72%), while 46 had a job (28%). Most of the participants had spent 8 years or more in education (153; 92%).

According to ICD-10 criteria, 57 patients were diagnosed with schizophrenia (34%), 4 with delusional disorder (2%); 10 with schizoaffective disorder (6%), 29 with bipolar disorder (18%), 27 with major depressive disorder (16%), 26 with personality disorder (16%), 12 with eating disorder (7%), and one with an anxiety disorder (1%). Mean age at illness onset was 29.6 (± 13.1) years. The mean number of previous hospitalizations was 6.4 (± 7.7).

### Principal components analysis

Item responses used the full range of scores (0–4) for each item, but many were highly skewed indicating that polychoric correlation coefficients were required [30,37-39]. The Kaiser-Meyer-Olkin measure verified the sampling adequacy for the analysis, KMO = 0.744. Nine items had correlations lower than .3, but none had high correlations (>.8). Diagonals of the anti-image correlation matrix were all above .5. Bartlett’s test of sphericity (*χ*^2^ (166) = 3024.3, *p* < .001), indicated that relations between items were sufficiently large for PCA.

Parallel analysis using 500 random correlation matrices permuted from the raw data [40] suggested three components should be retained (Table 2). After rotation, the items appeared to split into positively and negatively phrased (inversely scored) components. Cognitive improvement, plus an additional group learning item (item 23) appeared as a consistent component in all 3–6 component solutions. Of these, the 5-component solution was most promising as it explained 50.5% of the variance and had a better conceptual fit. Removal of low correlating items did not significantly improve the explained variance of the model whilst removal of seven inversely scored, highly skewed items resulted in a 5 component solution which explained 50.8% of the variance. This solution was selected as it provided better conceptual distinction between the previously defined components of sharing of emotions and experiences, cognitive improvement, group learning, difficulties in open expression and relationships.

**Table 2:** Parallel analysis results: eigenvalues greater than 1 and proportions of variance explained (N = 166)

**Variable**	**Eigenvalue**	**Mean of random eigenvalues**	**95 percentile of random eigenvalues**	**Proportion of variance**	**Cumulative proportion of variance**
**1**	**8.858**	**2.585**	**2.750**	**.227**	**.227**
**2**	**5.569**	**1.858**	**1.963**	**.143**	**.370**
**3**	**2.071**	**1.744**	**1.823**	**.053**	**.423**
4	1.637	1.657	1.724	.042	.465
5	1.557	1.585	1.641	.040	.505
6	1.300	1.523	1.584	.033	.538
7	1.231	1.461	1.516	.032	.570
8	1.117	1.407	1.452	.029	.599

Suggested number of variables to retain highlighted in bold.

Four items loaded highly (>.5) onto each component. PCA of these 20 items explained 60.8% of the variance. Orthogonal and oblique rotations were similar. As components were moderately correlated, the oblique rotation was preferred (Tables 3 and 4). Reliability analysis using Cronbach’s α on standardised values [37] demonstrated good reliability for all sub-scales and item totals (Table 5).

**Table 3:** Oblique rotation of 5 components with final 20 items and item reliability

		**Component 1: cognitive improvement**		**Component 2: group learning**		**Component 3: relationships**		**Component 4: difficulties in open expression**		**Component 5: sharing of emotions and experiences**	
**Item**	**Area**	**Pattern**	**Structure**	**Pattern**	**Structure**	**Pattern**	**Structure**	**Pattern**	**Structure**	**Pattern**	**Structure**
36. I listened to others carefully in the group	CI	**.898**	**.905**	.053	.254	.017	.163	-.021	.223	-.038	.028
30. I paid attention to what others said in the group	CI	**.872**	**.859**	-.161	.067	.071	.175	.042	.266	.015	.053
35. I expressed my thoughts clearly in the group	CI	**.505**	**.547**	.288	.374	-.313	-.096	.040	.186	.253	.278
33. I could remember what was said in the group	CI	**.462**	**.527**	.267	.384	-.145	.054	.049	.203	.224	.278
29. In the group, I realised how much my behavioural problems have improved	GL	-.205	-.011	**.727**	**.717**	.056	.244	.052	.099	.076	.225
19. I understood the reasons for my behaviour in the group	EE	.016	.190	**.665**	**.712**	.095	.295	.003	.107	.086	.239
21. In the group, I understood better how I usually deal with my problems	GL	.093	.257	**.605**	**.679**	.155	.344	-.010	.114	.057	.214
23. I learned how to manage good ineractinos with others in the group	GL	.235	.352	**.578**	**.571**	.127	.220	-.032	.074	**-.463**	-.310
9. I met new and positive people in the group	R	-.034	.091	.045	.246	**.759**	**.769**	-.027	.084	.036	.198
8. I built relationships of trust with others in the group	R	.065	.220	.124	.348	**.696**	**.763**	.044	.185	.078	.256
5. I was helped by others in the group.	R	-.087	.027	.109	.298	**.557**	**.632**	-.070	.033	.334	**.460**
6. I socialised with others in the group	R	.306	**.465**	.048	.268	**.520**	**.597**	.272	**.424**	-.119	.042
*18. I hid my feelings in the group	EE	.011	.217	-.199	-.059	.104	.187	**.877**	**.875**	.028	.094
*38. I found it difficult to express my thoughts clearly in the group	CI	.005	.258	.098	.195	.010	.156	**.871**	**.881**	-.047	.057
*16. I was afraid to express my opinion in the group	EE	.083	.294	-.057	.061	-.025	.103	**.853**	**.867**	.025	.094
*13. It was hard for me to talk about my problems in the group	EE	-.123	.122	.186	.225	-.101	.048	**.824**	**.798**	-.012	.075
2. I met people in the group who were experiencing the same problems as me	R	-.042	-.011	-.013	.126	.061	.189	-.065	-.002	**.705**	**.706**
1. Within the group I shared my personal experiences and life problems.	R	.193	.319	.124	.330	.077	.293	.186	.320	**.599**	**.669**
17. I talked with others about my suffering in the group	EE	.067	.144	.066	.253	.220	.365	-.028	.084	**.579**	**.639**
12. I was able to recognise my feelings in the group	EE	.057	.226	**.477**	**.615**	.136	.367	.050	.180	**.409**	**.541**
Eigenvalue		5.27		2.69		1.79		1.28		1.15	
Proportion of explained common variance (%)		26.3		13.4		8.9		6.4		5.8	
Reliability estimate+		.902		.830		.813		.928		.793	
Cronbach’s alpha		.742		.702		.753		.845		.725	
Cronbach’s alpha- standardised items		.743		.702		.759		.845		.730	

Absolute values greater than 0.4 shown in bold; High structure loading shown in grey.

EE- Emotional Expression; R- Relationships; GL- Group Learning; CI- Cognitive Improvement; H- Overall perceived helpfulness; * - Inversely scored item; + (Mislevy & Bock, 1990).

**Table 4:** Correlation matrix for oblique rotation of 5 component analysis

**Component**	**1: Cognitive improvement**	**2: Group learning**	**3: Relationships**	**4: Difficulties in open expression**	**5: Sharing of emotions and experiences**
**1: Cognitive Improvement**	1				
**2: Group Learning**	.230	1			
**3: Relationships**	.159	.271	1		
**4: Difficulties in open expression**	.266	.118	.147	1	
**5: Sharing of emotions and experiences**	.059	.199	.208	.095	1

**Table 5:** Proposed 5 subscales and item reliability

**Subscale and items**	**Mean**	**Standard deviation**	**Cronbach’s alpha**	**Cronbach’s alpha (standardised items)**
**1. Sharing of emotions and experiences**	8.13	3.00	.725	.730
1. Within the group I shared my personal experiences and life problems.				
2. I met people in the group who were experiencing the same problems as me.				
12. I was able to recognise my feelings in the group.				
17. I talked with others about my suffering in the group.				
**2. Cognitive Improvement**	10.02	2.56	.742	.743
30. I paid attention to what others said in the group.				
33. I could remember what was said in the group.				
35. I expressed my thoughts clearly in the group.				
36. I listened to others carefully in the group.				
**3. Group Learning**	8.19	2.82	.702	.702
19. I understood the reasons for my behaviour in the group.				
21. In the group, I understood better how I usually deal with my problems.				
23. I learned how to manage good interactions with others in the group.				
29. In the group, I realised how much my behavioural problems have improved.				
**4. Difficulty in open expression**	11.04	3.88	.845	.845
13. It was hard for me to talk about my problems in the group.				
16. I was afraid to express my opinion in the group.				
18. I hid my feelings in the group.				
38. I found it difficult to express my thoughts clearly in the group.				
**5. Relationships**	8.68	2.89	.753	.759
5. I was helped by others in the group.				
6. I socialised with others in the group.				
8. I built relationships of trust with others in the group.				
9. I met new and positive people in the group.				
**Scale Total**	**46.06**	**10.14**	**.849**	**.852**
Sum of above items				

## Discussion

Group interventions are widely provided in psychiatric settings, yet there is a paucity of research that focuses upon patients’ experiences of group sessions. In this study, a scale was developed that assessed group experiences of 166 patients with severe mental illness from three Italian community mental health centres. The Ferrara Group Experiences Scale (FE-GES) is relatively brief tool, consisting of 5 subscales with a total of 20 items which capture important and distinct aspects of patient experiences. It resulted from a systematic and conceptually driven development, and shows good psychometric qualities.

The five subscale components confirm factors identified in previous research and identify new aspects that invite further reflection. Below, the five subscale factors are described and their relationship to existing literature is briefly discussed.

### Sharing of emotions and experience

The experience of “sharing” with the therapist and other group members is regarded as one of the core elements to promote change in group treatments. In this subscale several existing factors are reflected: 1) sense of universality, 2) development of socializing abilities, 3) self understanding and 4) imitative behaviour [19]. As mentioned previously, universality, self-understanding, and cohesiveness have recently been indicated as the most important therapeutic factors [23]. Sharing of emotions and experiences among group members appears to be the core element of self-disclosure, suggested by Bloch and Crouch [22] as a driver of change. Sharing emotions and experiences may also contribute to the quality of the relational climate that Dierick and Lietaer described as a fundamental dimension of group therapies [28,41-43].

### Cognitive improvement

This subscale corresponds to Yalom’s concept of “imparting of information” [6,7]. It captures how cognitive abilities can be elicited and strengthened within the group process. Participants can improve their ability to focus, to remember what was said, to express thoughts in a clear way, and to listen. The items suggest that this improvement is connected to the relational aspect of listening and paying attention to statements of other patients, an intrinsic characteristic of group treatments.

### Group learning

The “Group learning” factor corresponds to research on group learning processes and reflects Yalom’s concepts of interpersonal learning and self understanding [6,7]. These concepts refer to developing a greater capacity to understand the rules and codes of relationships, achieving a higher level of self-awareness (self-understanding) and insight into the origins of problems and the unconscious motivations that underlie behaviours (“genetic insight”) [7]. Similar concepts are found in the work of MacKenzie [44] and Dierick and Lietaer [28], who by rearranging Yalom’s therapeutic factors, indicated the elements of “interpersonal learning” and “self-understanding” as an underlying dimension of psychological work.

### Difficulties in open expression

The conceptual development of the FE-GES has identified an aspect of group participation which, so far, has not been considered in group experience scales. The items of this subscale contrast with the therapeutic experiences described so far, indicating patients’ discomfort in talking about problems and communicating opinions and feelings. These obstacles to self- expression during group sessions may originate from therapists’ failure to build a safe and non-judgemental atmosphere, from the existence of strong cultural or social diversities within the group, or from the subjective characteristics of one or more members. In their study of inpatient psychotherapy groups, Leszcz, Yalom and Norden [24] suggested that the heterogeneous needs and capacities of patients may require a range of differing forms and structures in groups. When these difficulties arise, it is crucial that the therapist is aware of them so that these problems can be addressed. Such difficulties may undermine participation within the group and the success of intervention, by hampering the development of cohesion and self-disclosure [22]. This subscale may provide a means of identifying such difficulties during the therapy process and enable clinicians to adjust therapeutic programs to meet individual needs. It is important to note that these ‘negative’ experiences do not imply the absence of positive experiences, which would be captured by low scores on the other subscales. It is a distinct factor which may assist in improving group experiences of psychiatric patients.

### Relationships

This subscale has strong links to many of the proposed therapeutic factors in group psychotherapy research. Several of Yalom’s therapeutic factors are reflected: 1) Altruism, 2) Instillation of hope, 4) Development of socializing abilities, 5) Catharsis, and 6) “Corrective recapitulation of the primary family group”. The last factor supports reintegration and helps patients to accept insecure or maladaptive patterns of attachment which may have developed in infancy. Hence, it relates to attachment theory and can be particularly relevant to interventions utilising that theory. The items “I met new and positive people”, “I built relationships of trust with others” and “I was helped by others” may also be representative of the dimension that Dierick and Lieater referred to as “relational climate” [28].

### Limitations

The study has a number of limitations which should be taken into account. The limited sample size, from only one region of Italy means that results are not generalisable and will require further studies in different countries with larger samples. The measure was validated using a limited range of group treatments, each with differing approaches and levels of evidence to support their use in a clinical setting. It may therefore miss factors specific to a single modality of treatment. However, the application of the FE-GES across different modalities will enable comparisons to be made regarding the presence of common group factors.

As a consequence, the FE-GES should be cross-validated in other countries and in other services with different group treatments.

Finally, patients with a psychiatric diagnosis were not involved in the focus groups to generate items for the questionnaire but were involved subsequently to modify the content so that it was clear and easy to understand.

As a consequence, the FE-GES should be cross-validated in other countries and in other services with different group treatments.

## Conclusions

The subscales of the FE-GES are conceptually similar to other studies of group therapeutic factors. Relational and Cognitive dimensions are particularly linked to those originally identified by Yalom and subsequently described by other authors as key therapeutic factors in group therapy [6,7,22]. These results suggest a fundamental connection between the emotional and cognitive components of group experiences. Through relational and emotive group experiences cognitive learning is further advanced [24-27,45]. The questionnaire has good face and content validity. Further studies with existing scales are now needed to confirm concurrent validity.

In summary, our preliminary validation indicates that the FE-GES appears a useful tool in the assessment of group treatment experiences of patients with severe mental illnesses. It is designed to detect distinct and typical factors of group experiences, and can be used to assess mediating processes and outcomes in research. Most importantly, it is very brief which is essential for a scale to be administered in routine care. The FE-GES can therefore be used in both research and in the evaluation and quality management of group treatments in routine care. It may aid not only clinical decision making and supervision, but also contribute to the further development of group therapy theory.

## Competing interests

The authors declare that they have no competing interests.

## Authors’ contributions

LG, BB, MR and SP wrote the proposal. RC, BB, LM, LB and MM participated in data collection. RC, BB and CC wrote the manuscript. CC analyzed the data. MR, LG and SP approved the proposal with some revisions and supervised the final paper. All authors read and approved the final manuscript.

## Pre-publication history

The pre-publication history for this paper can be accessed here:

http://www.biomedcentral.com/1471-244X/13/242/prepub
